# Meeting of the New York State Medical Society

**Published:** 1877-06

**Authors:** 


### Meeting of the New York State Medical Society.

It will be remembered that change was made in the time of meeting of the New York State Medical Society, at its last meeting. The Society adjourned to meet at 11 A. M., on the third Tuesday of June,—June 19th, 1877. The meetings of the State Medical Society have always been made instructive and entertaining, and physicians of New York will be interested the transactions as well as in the social opportunities thus afforded. Permanent members and delegates should not fail to attend.

———:o:———

